# Bread Poison

**Published:** 1876-02

**Authors:** 


### BREAD POISON.

OF all kinds of rickety things, a rickety body is about the worst: its prominent features being a big head, with nothing in it, bowed legs, spindle shins, and club joints; the right shoulder rises, the left shoulder falls, the breast-bone projects, the back-bone twists, and the poor little pot-bellied, knock-kneed, tottering, pale-faced creature, divides our sympathy and our disgust. Cold, and hunger, and dirt, and neglect, entail this disease on many children of the poor; but the same disease is sometimes observable in persons of the middle and higher classes of society. Medical men have turned their attention in this direction, and in tracing out probable causes, have ascertained this general fact beyond a peradventure—that rickets most prevail in those localities, and among those families, other things being equal, where baker's bread is most used.

*The London Lancet*, for July, 1875 has taken up the subject; and Dr. Snow found several hundred grains of alum in four pounds of bread. And as a child will easily eat a pound of bread in a day, especially when it is its chief food, and as thirty grains of alum is a speedy emetic, we cannot wonder that terrible effects should result, in time, from a child's eating two or three ounces of alum every week of its life. The immediate effect of the alum is to destroy that principle in the flour which strengthens the bones; and the same as to grown persons. And what other ill effects may result from the habitual use of alumed bread, we do not know; but if Dr. Snow's general facts are true, we had better allow the bakers to eat their own bread, and we make bread for ourselves, and let us eat food, not physic.

Alum makes the bread appear whiter and lighter, at the same time. There is more water in a loaf which has alum than in one which has none, consequently, the unalumed bread, pound for pound, is the cheapest. No one will contend that alum is either nutritious or healthful, and although it will not give the rickets to all who use it in their bread; nor will all escape the rickets who do not use baker's bread, with alum in it—it is clearly the dictate of wisdom for every family to do its own cooking, and dismiss instantly every cook who is not a good bread-baker.

If there is much alum in the bread, a piece of it put in water with some logwood in it will turn the water to a dark color. The indigestibility of London bakers' bread is the common remark of foreigners, and as alum is so universally used, Liebig thus accounts for the fact. We may then reasonably conclude, that one cause of dyspepsia is the free use of Alumed Bread.

